# Retraction: LncRNA ZEB2-AS1 regulates the drug resistance of acute myeloid leukemia *via* the miR-142-3p/INPP4B axis

**DOI:** 10.1039/d1ra90063c

**Published:** 2021-02-01

**Authors:** Laura Fisher

**Affiliations:** Royal Society of Chemistry Thomas Graham House, Science Park, Milton Road Cambridge CB4 0WF UK advances-rsc@rsc.org

## Abstract

Retraction of ‘LncRNA ZEB2-AS1 regulates the drug resistance of acute myeloid leukemia *via* the miR-142-3p/INPP4B axis’ by Kai Wang *et al.*, *RSC Adv.*, 2019, **9**, 39495–39504, DOI: 10.1039/C9RA07854A.

The Royal Society of Chemistry hereby wholly retracts this *RSC Advances* article due to concerns with the reliability of the data. The images in the article, and raw data provided by the authors, were screened by an image integrity expert. The raw data provided by the authors was found to closely resemble raw data for a number of other articles, which is unexpected given that there are completely different author lists for these articles.

In addition, the paper was analysed by experts who fact-checked the identities of the described nucleotide sequence reagents,^1^ and found errors with the following nucleotide sequence reagents reported in the article: ZEB2-AS1 forward and reverse primers and miR-142-3p reverse primer. The claimed identities of the nucleotide sequences for the reported primers are incorrect and, therefore, the results shown in Fig. 1–6 are unreliable.

Given the significance of the concerns about the validity of both the data in the article and the raw data provided by the authors, the findings presented in this paper are not reliable.

Kai Wang opposes the retraction. The other authors have been informed but have not responded to any correspondence regarding the retraction.

Signed: Laura Fisher, Executive Editor, *RSC Advances*

Date: 19^th^ January 2021

## Supplementary Material
